# Assessment of primary rehabilitation needs in neurological rehabilitation: translation, adaptation and face validity of the Danish version of Rehabilitation Complexity Scale-Extended

**DOI:** 10.1186/s12883-016-0728-7

**Published:** 2016-10-21

**Authors:** Thomas Maribo, Asger R. Pedersen, Jim Jensen, Jørgen F. Nielsen

**Affiliations:** 1 Department of Public Health, Section of clinical social medicine and rehabilitation, Aarhus University, Aarhus, Denmark; 2 DEFACTUM, Central Denmark Region, Aarhus, Denmark; 3 Hammel Neurorehabilitation and Research Centre, Aarhus University, Hammel, Denmark

**Keywords:** Assessment of rehabilitation needs, Translation and adaption, Validation, Complexity of rehabilitation needs, Neurological rehabilitation, Acquired brain injury, Needs assessment, Psychometric properties, Rehabilitation Complexity Scale-Extended

## Abstract

**Background:**

Assessing primary rehabilitation needs in patients with acquired brain injury is a challenge due to case complexity and the heterogeneity of symptoms after brain injury. The Rehabilitation Complexity Scale-Extended (RCS-E) is an instrument used in assessment of rehabilitation complexity in patients with severe brain injury. The aim of the present study was to translate and test the face validity of the RCS-E as a referral tool for primary rehabilitation. Face validity was tested in a sample of patients with acquired brain injury.

**Methods:**

Ten clinicians and records from 299 patients with acquired brain injury were used in the translation, cross-cultural adaptation and face validation study of the RCS-E. RCS-E was translated into Danish by a standardized forward-backward translation by experts in the field. Face validity was assessed by a multi-professional team assessing 299 patients. The team was asked their opinion on whether the RCS-E presents a sufficient description of the patients.

**Results:**

The RCS-E was translated according to international guidelines and tested by health professionals; some adaptations were required due to linguistic problems and differences in the national health system structures.

The patients in the study had a mean age of 63.9 years (SD 14.7); 61 % were male.

We found an excellent face validity with a mean score of 8.2 (SD 0.34) assessed on a 0–10 scale.

**Conclusions:**

The RCS-E demonstrated to be a valid assessment of primary rehabilitation needs in patients with acquired brain injury. Excellent face validity indicates that the RCS-E is feasible for assessing primary rehabilitation needs and the present study suggests its applicability to the Danish health care system.

**Electronic supplementary material:**

The online version of this article (doi:10.1186/s12883-016-0728-7) contains supplementary material, which is available to authorized users.

## Background

After acute treatment, most patients with acquired brain injury will continue rehabilitation according to the complexity of their injury [1]. Assessing primary rehabilitation needs, i.e., for the rehabilitation starting immediately after acute treatment, is a challenge due to case complexity and the heterogeneity of symptoms after brain injury [2]. Complexity relates to the number of different factors that affect the course of rehabilitation, which has traditionally been evaluated in terms of comorbidity or physical dependency [3, 4]. The integrative biopsychosocial model (ICF) places function between health and contextual factors, making complexity much more important than comorbidity [5]. Physical dependency does not capture needs for specialist medical care, specialist nursing care, or the need for cognitive, behavioural or other psychological interventions. Frequently used outcome measures in neurological rehabilitation such as the Barthel Index and the Functional Independence Measure evaluate independence and physical dependency, making them unfit for assessing complexity and as referral tools [3].

In rehabilitation, variables from different domains usually interact in a non-linear way, with complicated interrelationships that impede assessment of rehabilitation needs and call for multi-professional assessment [3].

Assessing the complexity of primary rehabilitation needs in order to refer patients to the appropriate care setting is a worldwide challenge [3, 6]; only few tools are concerned with rehabilitation complexity; all have their limitations. An editorial review from 2011 gave examples of four tools [3]. One of the recommended tools was the Rehabilitation Complexity Scale (RCS) [3]. The RCS was introduced in 2007 as a measure of case-load complexity in rehabilitation [7], developed to detect the clinical need for higher-level services instead of local services; differentiating between ‘complex specialized’ and ‘district specialist’ rehabilitation services is found to be valid [7]. Since the editorial review in 2011, the RCS has undergone further development [8]. The earlier versions of the RCS had problems with ceiling effects, and no information on the need for special equipment was collected. Furthermore, the earlier versions did not capture the “Risk” or needs for supervision of patients who were mobile, but confused; e.g., in cognitive behavioural rehabilitation settings [8]. The Rehabilitation Complexity Scale-Extended (RCS-E) was developed to address these problems [8]. The RCS-E has proved reliable [9, 10] and is used as a measure of complexity within the rehabilitation process, especially in neurological rehabilitation [3, 11, 12].

The aim of this study was to formally translate and cross-culturally adapt the RCS-E into Danish and to test its face validity. It was hypothesized that the translated RCS-E would have a high face validity of >7 scored on a 0–10 point scale.

## Methods

### Outcome measure

The RCS-E consists of six domains shown in Table 1 [8]. The first two domains are usually scored as care *or* risk, leaving a scale with five domains.

**Table 1:** The domains in the Rehabilitation Complexity Scale-Extended

Abbreviation	Domain	Range
C	Basic care (support needs)	0–4
R	Risk (cognitive or behavioural needs)	0–4
N	Skilled nursing needs	0–4
M	Medical needs	0–4
Therapy needs		
TD	Required number of different therapy disciplines	0–4
TI	Therapy intensity	0–4
E	Equipment needs	0–2

### Translation and cross-cultural adaptation

The translation and cross-cultural adaptation of the RCS-E to create a Danish version was done according to internationally accepted guidelines [13] and with permission from the developers of the RCS-E. The group responsible for the translation consisted of seven persons. The five forward translators (native Danish speakers) included a physiotherapist (PT), an occupational therapist (OT), a medical doctor (MD), and two nurses (RN). The two first authors (PT and OT) translated the entire RCS-E, the MD translated “medical needs” and “risk”; two RNs translated “basic care” and “skilled nursing needs”. The different versions were synthesized into a forward version. The forward translation was discussed by three groups of health care professionals (MD, RN and OT/PT) working with specialized neurorehabilitation. Each group discussed only the domains they were to score: “medical needs” and “risk” were discussed by a group of MDs, “basic care” and “skilled nursing needs” were discussed by a group of RNs, and the required number of different therapy disciplines, therapy intensity, and equipment needs were discussed by a group of OTs/PTs. Comments from this process were incorporated in the final forward translation. The backward translations were done by two English native speakers, one with a background in rehabilitation, the other a non-medical professional translator. The two backward translations were synthesized by the first author and sent to the developer of the RCS-E for approval.

To check acceptability and comprehension a pilot-test was carried out with 25 patients.

### Study design for face validity

Data from 300 consecutive patients with acquired brain injury aged 18 or more and admitted to Hammel Neurorehabilitation and Research Centre, Denmark, between February and August 2014, was used in the test for face validity.

The RCS-E was scored by an expert team comprising a MC, a RN and an OT not involved in the translation process. Each domain was scored by a single team member; the MD scored “medical needs” and “risk”, the RN scored “basic care” and “skilled nursing needs”, and the OT scored “required number of different therapy disciplines”, “therapy intensity”, and “equipment needs”. After a pilot phase where the team used 10 medical records to jointly score the RCS-E, each team member scored their own domains. In cases of doubt, the relevant records would be discussed in the team.

Face validity is one of the basic psychometric requisites for an assessment tool and addresses whether the scale appears to actually cover the concept it intends to measure [14]. It considers the relevance of a test as it appears to testers: a test can be said to have face validity if it “looks like” it will in fact measure what it is supposed to measure [15].

Face validity is desirable as tools that are perceived as irrelevant may be answered with less care, making them less reliable. Face validity is evaluated by a subjective judgment of experts [14].

After every 10 patients, each team member was asked two questions: 1) “Did you have any problems scoring one of the 10 latest patients? – If yes, indicate which patient(s)”, and 2) “On average over the last 10 patients, does the RCS-E present a sufficient description of the patient in the areas you have been asked to assess? Please indicate on a scale from 0 to 10, where 0 is not at all sufficient and 10 is sufficient.” Case material was collected from medical records.

Hammel Neurorehabilitation and Research Centre is a rehabilitation hospital, treating patients with acquired brain injury, and has a background population of 2.9 million individuals. Patients with severe acquired brain injury are referred for inpatient rehabilitation after treatment at intensive care units or departments of neurology or neurosurgery.

Depending on the patient’s clinical severity at admission, two multidisciplinary rehabilitation options were available: 1) complex specialized service: High-intensity rehabilitation and therapy during all waking hours; carried out by staff experienced in neurorehabilitation of severely affected patients, and 2) district specialist service: Moderate-intensity rehabilitation and therapy carried out only during daytime hours (until 6 pm).

### Statistical analysis

Descriptive statistics were used for age, gender, diagnosis and whether experts observed problems scoring the RCS-E. The experts’ scoring on face validity was presented as mean, standard deviation (SD) and range.

The COSMIN checklist suggests >100 as the number required for assessment of structural validity [16]. As we were to test 300 cases for another study (Pedersen AR, Nielsen JF, Jensen J, Maribo T. Referral decision support in patients with subacute brain injury: evaluation of the Rehabilitation Complexity Scale - Extended. Disabil Rehabil. 2016. Jul 6:1-7. Epub ahead of print), this number was chosen as the sample size.

There were no missing data: all data were collected electronically, and respondents were not given the possibility of continuing if an answer was missing.

## Results

### Translation and cross-cultural adaptation

The forward translation from English into Danish revealed certain cross-cultural and linguistic issues. The domains “risk” and “therapy needs” in particular gave rise to questions. This became evident through the pilot test, prompting the need to reword several items. A number of MDs, RNs, nursing assistants, OTs, and PTs participated in the pilot test, which included 25 patients.

The English word “therapy” changes meaning when translated as the Danish “terapi”: the Danish term refers almost exclusively to physiotherapy or occupational therapy. In sections TD and TI *Therapy needs* are translated as *interdisciplinary interventions*, and *therapy disciplines* translated as *professions.*

The risk section refers to *Mental Health Act* (R2 and R3). There is no such act in Denmark and thus no additional paperwork. The reference was removed.

In the therapy section, a reference is made to The Northwick Park Therapy Dependency Assessment (NPTDA). NPTDA is not translated into Danish, and the reference was removed.

**Fig. 1:**
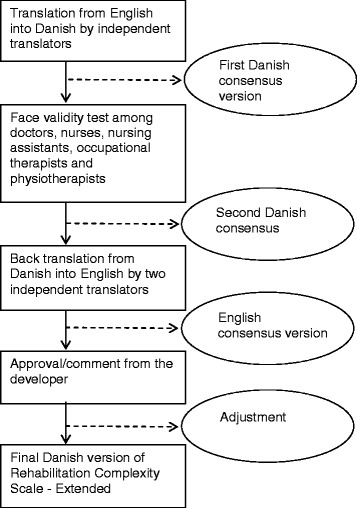
Translation of the Rehabilitation Complexity Scale - extended

The adjusted version was then back translated, and this version was approved by Lynne Turner-Stokes. The procedure only gave rise to minor changes; for example, the translation of *Environmental control* (E2) was changed. Figure 1 shows the translation and adaptation process. The Danish version of RCS-E is available as Additional files 1 and 2 give directions to the English Version of RCS-E.

Three hundred records were included, but due to a mistake one record appeared in duplicate, leaving 299 included patients. Demographic details and clinical characteristics are presented in Table 2.

**Table 2:** Demographics and clinical characteristics at admission

Age [years]	63.88 (14.7)
Sex, male/female	181 (61 %)/118 (39 %)
Diagnosis	
Acquired brain injury	
Vascular (stroke, SAH)	246 (82 %)
Traumatic	24 (8 %)
Other (e.g., Hypoxic/inflammatory)	24 (8 %)
Guillain-Barré and other peripheral neuropathies	5 (2 %)

Values are mean (SD) or n (percentage); *n* = 299

There were no missing items in the baseline information data included in the analysis of face validity.

A summary of the distribution of RSC-E scores within each domain, and the total scores are presented in Table 3.

**Table 3:** (*n* = 299)

Domain	Median [IQR] (range)
Basic care (support needs)	1 [1; 2] (0; 4)
Risk (cognitive or behavioural needs)	1 [1; 2] (0; 3)
Skilled nursing needs	2 [2; 3] (0; 4)
Medical needs	1 [1; 2] (0; 3)
Required number of different therapy disciplines	2 [2; 3] (2; 4)
Therapy intensity	2 [2; 2] (1; 4)
Equipment needs	1 [1; 1] (0; 2)
Total RCS-E	10 [9; 12] (5; 21)

The team members had difficulties scoring 11 records (3.7 %). The MD and the RN had problems scoring one patient each, while the OT had problems scoring 9 records. These records were discussed by the team.

Overall the team members were pleased with the RCS-E. The team gave a mean score of 8.2 SD 0.34 in their answers to the question “On average over the last 10 patients, does the RCS-E present a sufficient description of the patient?” See Table 4.

**Table 4:** Face validity for the Rehabilitation Complexity Scale-Extended

Team member	Medical doctor	Nurse	Therapist
Domains scored	(M and R)	(C and N)	(TD, TI and E)
Mean (SD)	8.4 (0.49)	7.8 (0.38)	8.3 (0.91)
Range	[8, 9]	[7, 8]	[6, 10]

*C* basic care (support needs), *R* risk (cognitive or behavioural needs), *N* skilled nursing needs, *M* medical needs, *TD* required number of different therapy disciplines, *TI* therapy intensity, *E* equipment needs. *After assessing every 10 patients, each team member answered the question: “On average over the last 10 patients, does the RCS-E present a sufficient description of the patient in the areas you have been asked to assess? Please indicate on a scale from 0 to 10, where 0 is not at all sufficient and 10 is sufficient”*

## Discussion

The RCS-E was successfully translated and adapted into Danish. High face validity was indicated by all team members; only few records (3.7 %) needed discussion in the team.

The translation from English into Danish and back was carried out according to international guidelines [13]. In this process, small adaptations to the original English version were made, such as excluding the Mental Health Act referenced in the original.

The team carried out preliminary training by discussing the RCS-E and scoring 10 patients together in the team. This pilot test and discussion process took five hours, and after this initial trial the team felt ready to use the RCS-E; no further training was needed.

The RCS-E proved to be applicable to almost all the records examined: the team only found difficulties in assessing 11 (3.7 %) of the records. This confirmed our hypothesis that <10 % of the cases would be difficult to score.

After a short discussion on the records that were difficult to score, the team was able to score these cases. Typically, the problems concerned therapy intensity as the medical records lacked information on whether the presence of an assistant was necessary, making it difficult to distinguish between TI2 and TI3. Another problem was to determine the required number of disciplines in cases where it was difficult to distinguish between TD2 and TD3. Almost all patients needed occupational therapy and physiotherapy, but in the acute state it was in some cases difficult to determine whether an additional one or two disciplines were needed. The discussion on these records showed that the problems were due to missing information in the records, meaning that the problem did not reside with the RCS-E as such, but with the records.

The inclusion of 299 cases in the face validity test exceeds the sample size suggested by the COSMIN group and Terwee et al. [16, 17].

The present study has some limitations as the team consisted only of persons from a single rehabilitation institution. Involving more teams and other sites using the RCS-E could have strengthened the result.

There is no consensus on how to interpret results from tests of face validity on a 0–10 point scale. Thus, we chose a score of >7 to indicate high validity. We believe a mean score of 8.2 with a narrow SD is satisfying and indicates high face validity.

As we did not aim for a full evaluation of content validity it is not possible to fulfil all criteria in the COSMIN group checklist “Content validity (including face validity)” [18], but more than 10 different health professionals were involved in the translation process (primarily during the pilot testing) and the test of face validity, and all indicated that the RCS-E was useful as a tool for assessment of primary rehabilitation needs. This supports the good face validity found in this study. The developer of the RCS-E and an Italian group carrying out similar work also report no problems in scoring RCS-E, supporting the case for good face validity [8–10, 19].

It has been suggested that care or risk should be assessed [8]. As the RCS-E is a multi-professional assessment, assessing both care and risk might be worth considering. In this study, care was assessed by the RN and risk by the MD. The expert team found that assessing both aspects was a rational move, as this provided a more comprehensive picture of the patient.

Our aim was to test whether a referral support tool used for referral for primary rehabilitation in patients needing ‘complex specialized’ or ‘district specialist’ rehabilitation services in the UK could be used in other countries; this aim was met.

## Conclusions

We have successfully translated and adapted the RCS-E into Danish, and the Danish version demonstrates excellent face validity. Moreover, the face validity presented provide more credibility to the use of RCS-E, as a tool for assessing complexity and as decision aid in the referral process. Assessing primary rehabilitation needs and better referral is crucial as intensive neurological rehabilitation is expensive. Compared to the regular clinical approach a systematic assessment of the elements in the RCS-E in combination with evaluation of the personal and contextual factors of the patient could lead to improvement in the referral process. Recognizing the complexity in assessment of primary rehabilitation needs and using variables from different domains is important [3] and the RCS-E is an easy and quick tool to use in the process.

Further studies should test other psychometric properties of the RCS-E, primarily whether it can distinguish between patients’ needs for primary rehabilitation.

## Additional files

Additional file 1: The Danish version of RCS-E. (PDF 68 kb)
Additional file 2: How to assess the English version of RCS-E. (PDF 100 kb)
Additional file 3: Data from face validity study. The dataset has 10 variables: Variable a: *Case_no* refers to the case numbers; Variables b, e, h: **_problems* answers to question “Did you have any problems scoring one of the 10 latest patients?”; Variables c, f, i: **_problem_ID* gives the ID of the case with a problem; Variables d, g, j: ** _score* answers to question “On average over the last 10 patients, does the RCS-E present a sufficient description of the patient in the areas you have been asked to assess? Please indicate on a scale from 0 to 10, where 0 is not at all sufficient and 10 is sufficient”. (CSV 1 kb)

## Abbreviations

MD: Medical doctor
OT: Occupational therapist
PT: Physiotherapist
RCS: Rehabilitation Complexity Scale
RCS-E: The Rehabilitation Complexity Scale-Extended
RN: Nurse
SD: Standard deviation

## References

[1] Intercollegiate Stroke Working Party (2012). National clinical guideline for stroke: fourth edition.

[2] National Institute for Health and Care Excellence (2013). NICE clinical guideline 162. Stroke rehabilitation: long-term rehabilitation after stroke.

[3] Wade D (2011). Complexity, case-mix and rehabilitation: the importance of a holistic model of illness. Clin Rehabil.

[4] Turner-Stokes L, Sutch S, Dredge R, Eagar K (2012). International casemix and funding models: lessons for rehabilitation. Clin Rehabil.

[5] WHO, World Health Organization (2001). ICF - International classification of functioning, disability and health.

[6] Poulos CJ, Magee C, Bashford G, Eagar K (2011). Determining level of care appropriateness in the patient journey from acute care to rehabilitation. BMC Health Serv Res.

[7] Turner-Stokes L, Disler R, Williams H (2007). The Rehabilitation Complexity Scale: a simple, practical tool to identify ‘complex specialised’ services in neurological rehabilitation. Clin Med.

[8] Turner-Stokes L, Scott H, Williams H, Siegert R (2012). The Rehabilitation Complexity Scale--extended version: detection of patients with highly complex needs. Disabil Rehabil.

[9] Turner-Stokes L, Williams H, Siegert RJ (2010). The Rehabilitation Complexity Scale version 2: a clinimetric evaluation in patients with severe complex neurodisability. J Neurol Neurosurg Psychiatry.

[10] Roda’ F, Agosti M, Corradini E, Lombardi F, Maini M, Brianti R. Cross-Cultural adaptation and preliminary test-retest reliability of the Italian version of the Complexity Rehabilitation Scale-Extended (13th Version). Eur J Phys Rehabil Med. 2014;51:439-46. 24621987

[11] Troigros O, Bejot Y, Rodriguez PM, Shoaib F, Ellis H, Wade D (2014). Measuring complexity in neurological rehabilitation: the Oxford Case Complexity Assessment Measure (OCCAM). Clin Rehabil.

[12] Turner-Stokes L, Sutch S, Dredge R (2012). Healthcare tariffs for specialist inpatient neurorehabilitation services: rationale and development of a UK casemix and costing methodology. Clin Rehabil.

[13] Beaton DE, Bombardier C, Guillemin F, Ferraz MB (2000). Guidelines for the process of cross-cultural adaptation of self-report measures. Spine (Phila Pa 1976).

[14] Keszei AP, Novak M, Streiner DL (2010). Introduction to health measurement scales. J Psychosom Res.

[15] Portney LG, Watkins MP. Foundations of clinical research applications to practice, 3rd edn. Upper Saddle River: Pearsons; 2007.

[16] Terwee CB, Mokkink LB, Knol DL, Ostelo RW, Bouter LM, de Vet HC (2011). COSMIN checklist with 4-point scale.

[17] Terwee CB, Bot SD, de Boer MR, van der Windt DA, Knol DL, Dekker J (2007). Quality criteria were proposed for measurement properties of health status questionnaires. J Clin Epidemiol.

[18] Terwee CB, Mokkink LB, Knol DL, Ostelo RW, Bouter LM, de Vet HC (2012). Rating the methodological quality in systematic reviews of studies on measurement properties: a scoring system for the COSMIN checklist. Qual Life Res.

[19] Galletti L, Benedetti MG, Maselli S, Zanoli G, Pignotti E, Iovine R. Rehabilitation Complexity Scale: Italian translation and transcultural validation. Disabil Rehabil. 2015: 1–10. doi: 10.3109/09638288.2015.1024340. 10.3109/09638288.2015.1024340 25875050

