# Supplementary material for: Assessment of primary rehabilitation needs in neurological rehabilitation: translation, adaptation and face validity of the Danish version of Rehabilitation Complexity Scale-Extended
Source: BMC Neurol. 2016 Oct 21;16:205. doi: 10.1186/s12883-016-0728-7 (PMC5073960; doi:10.1186/s12883-016-0728-7)
Supplement: Additional file 1: — The Danish version of RCS-E. (PDF 68 kb) [file 12883_2016_728_MOESM1_ESM.pdf]

RCS-E

The Rehabilitation Complexity Scale: extended (DK1)

**PATIENTOPLYSNINGER**<br>**Navn:**<br>**Dato: …../…../…….**<br>**CPR:**<br>**Sæt ring om det højeste gældende niveau for hver kategori**	
**BEHOV FOR HJÆLP TIL GRUNDLÆGGENDE PLEJE**<br>Omfatter hjælp til grundlæggende plejeaktiviteter (enten fysisk hjælp eller standby opsyn)<br>Herunder f.eks. bad/vask, påklædning, personlig hygiejne, toiletbesøg, spisning, basal egenomsorg	
**C 0**	**Stort set selvhjulpen.** Klarer grundlæggende pleje stort set uden hjælp.<br>Kan ind imellem have brug for hjælp til at lægge ting frem eller afslutte opgaver, eks. påsætning af skinne, binde snørebånd eller lignende.
**C 1**	Har behov for **hjælp fra 1 person** til de fleste former for grundlæggende pleje, dvs. påklædning, personlig hygiejne osv. Kan ind imellem have brug for hjælp fra 2 personer til enkelte ting, f.eks til bad.
**C 2**	Har behov for **hjælp fra 2 personer** til hovedparten af den grundlæggende pleje.
**C 3**	Har behov for hjælp fra **3 personer** til grundlæggende pleje.
**C4**	Kræver konstant **1:1 opsyn,** f.eks for at håndtere konfusion og sikre patientens sikkerhed
**RISIKO – KOGNITIVE/ADFÆRDSMÆSSIGE BEHOV**<br>Inkluderer nødvendige opsyn i forhold til patientens sikkerhed eller i forhold til at håndtere konfusion, eksempelvis hos patienter med tendens til at vandre rundt eller hos patienter med adfærdsmæssige eller neuropsykologiske problemer.	
**R0**	**Ingen risiko** – i stand til at varetage egen sikkerhed og kan færdes alene ude og inde.<br>I stand til at varetage egen sikkerhed
**R1**	**Lav risiko** – standard-forholdsregler, let overvågning i et beskyttet miljø, men har behov for hjælp udenfor det beskyttede miljø.<br>Klarer egen sikkerhed i et beskyttet miljø, behøver kun rutinecheck, men har behov for ledsagelse og hjælp udenfor det vante/beskyttede miljø.
**R2**	**Mellemhøj risiko** – ekstra sikkerhedsforanstaltninger er påkrævet.<br>Ekstra sikkerhedsforanstaltninger påkrævet, selv indenfor et beskyttet miljø; f.eks. alarmer, sensorer eller monitorering, der er mere omfattende end standard (f. eks. check hver eller hveranden time).
**R3**	**Høj risiko** – skal observeres hyppigt<br>Behov for hyppig observation, selv i et beskyttet miljø – f.eks. check 1-2 gange hver time, eller 1:1 opsyn dele af dagen eller natten.
**R4**	**Meget høj risiko** – behov for konstant 1:1 opsyn<br>Behov for 1:1 opsyn hele tiden.

**SYGEPLEJEFAGLIGE BEHOV**<br>Beskriver behovet for sygeplejefaglige ydelser fra sygeplejersker eller fra sygeplejersker med specialoplæring indenfor neurosygepleje.	
**N 0**	Intet behov for ydelser fra en **uddannet sygeplejerske** – behovene kan dækkes af en assistent
**N 1**	Har behov for en **uddannet sygeplejerske** (med almene plejemæssige færdigheder). Eks. medicinering, sår/stomipleje, plejeobservationer, sondeernæring, etablering af IV-infusioner, etc.)
**N 2**	Kræver intervention fra plejepersonale, som er oplært i, og har erfaring med neurorehabilitering.<br>Eks. til at gennemføre et lejringsprogram, øve gang/stand, påsætte skinner og til psykologisk støtte.
**N 3**	Har behov for **højt specialiseret sygepleje** Eks. til meget komplekse behov som<br>• Håndtering af tracheostomi, håndtering af krævende adfærd/psykoser/komplekse psykologiske behov.<br>• Meget komplicerede posturale, kognitive eller kommunikative behov.<br>• Vegetative eller minimalt bevidste responderende stadier, locked-in syndromer.
**N 4**	Høj grad af afhængighed af specialiserede sygeplejersker (kræver højt specialiserede plejefærdigheder og intensiv pleje)<br>Ofte medicinsk ustabile, kræve hyppig monitorering/intervention fra kvalificeret pleje - hver time eller oftere (kræver ofte også specialuddannelse indenfor eks. administrering af IV medicin, ventilering etc.)
**LÆGEFAGLIGE BEHOV**<br>Beskriver det omtrentlige behov for lægelig (medicinsk/kirurgisk) behandling	
**M 0**	**Ingen aktiv medicinsk indgriben**<br>(Kan håndteres af patientens praktiserende læge ved almindelige løbende besøg)
**M 1**	**Basal undersøgelse/tilsyn/behandling**<br>(Der kræver ikke-akut hospitalsbehandling, kan udføres på hospital uden læger i døgnbemanding).<br>Eks. kræver kun standard blodprøve/-scanning. Medicinsk monitorering kan håndteres ved gennemgang ved reservelæge 2 -3 x ugentligt med standardkonsultation ved stuegang + telefonopkald ved behov.
**M 2**	**Specialiseret medicinsk/psykiatrisk indgriben mhp. diagnose eller behandling** (Der kræver hospitalsindlæggelse).<br>Eks. kræver mere komplekse undersøgelser eller specielle medicinske faciliteter såsom dialyse, respiratorisk støtte. Jævnligt eller uforudsigeligt behov for konsultation eller medicinsk specialistrådgivning, kirurgisk intervention, psykiatrisk evaluering/behandling.
**M 3**	**Potentielt ustabil medicinsk/psykiatrisk tilstand.** Behov for 24 timers akut medicinsk/psykiatrisk behandling (afhængig af behov).<br>Potentielt ustabil: kan kræve intervention på alle tider af døgnet, eks. ved ukontrollerede epileptiske anfald, svækket immunforsvar – eller justering af psykiatrisk medicin/risikovurdering i nødstilfælde etc.. Har behov for at blive håndteret i et miljø med 24 timers tilstedeværelse af medicinsk/psykiatrisk dækning.
**M 4**	**Akut medicinsk/kirurgisk problem (eller psykiatrisk krise).** Behov for intervention på alle tider af døgnet.<br>Behov for akut medicinsk/kirurgisk håndtering af eks. infektioner, akutte komplikationer, post-kirurgisk behandling, dvs. behov for medicinsk (eller kirurgisk eller psykiatrisk) overvågning/behandling døgnet rund, hvadend det er planlagt eller ikke planlagt.

BEHOV FOR ØVRIGE TVÆRFAGLIGE INDSATSER

Beskriv:

a) antallet af forskellige faggrupper, der er behov for, og
b) intensiteten af indsatsen.

Inkluderer individuelle eller gruppebehandling som gennemføres af øvrige faggrupper, men IKKE rehabiliteringsinput fra plejepersonalet, som er scoret i N2.

Discipliner: Angiv antallet af forskellige faggrupper der er aktivt behov for i behandlingen

**TD 0**	**0**	**Marker de faggrupper, der er involveret:**		
**TD 1**	**1** faggruppe	Fysioterapeut<br>Ergoterapeut<br>Talepædagog<br>Diætist<br>Socialrådgiver<br>Psykolog	Pædagog<br>Lærer<br>Bandagist<br>Musik-/<br>kunstterapeut	Andre:
**TD 2**	**2-3** faggrupper			
**TD 3**	**4-5** faggrupper			
**TD 4**	**≥6** faggrupper			

Intensitet: Angiv det **overordnede intensitetsniveau for** den **faglige indsats,** som kræves af det samlede team (i forhold til faggrupper nævnt under TD)

**TI 0**	**Ingen indsats.**<br>(eller<1 time i alt pr. uge – rehabiliteringsbehovet opfyldes af sygepleje/plejepersonale eller eget træningsprogram)
**TI 1**	**Lav intensitet – mindre end daglig behandling** (f.eks. undersøgelse/revurdering/vedligeholdelse/opsyn) **ELLER Kun gruppebehandling.**<br>Dvs. patienten modtager ikke behandling hver dag (eller har < 1 time per dag); dette betyder normalt at a) patienten aktuelt overvejende har brug for pleje, sygepleje eller medicinsk behandling, eller b) er på et lav-intensitet revurderingsophold eller gruppebehandling – eller c) er i gang med en nedtrapning af behandlingen som forberedelse til udskrivelse.
**TI 2**	**Moderat – daglig indsats** – individuelle behandlinger med en behandler til de fleste behandlinger **ELLER meget intensive gruppebehandlinger ≥ 6 timer dagligt**<br>Dvs. patienten kan modtage behandling af flere forskellige faggrupper (se TD), men behandles af én behandler af gangen. Patienten vil normalt modtage behandling hver dag, fem dage om ugen, totalt 2-3 timer dagligt (hvor noget af tiden kan være selvtræning eller superviseret på afstand hvis muligt).<br>Eller de har gruppebehandling på høj-intensivt niveau (>6 timer dagligt).
**TI 3**	**Højt niveau – daglig indsats med behandler plus assistent og/eller gruppebehandling.**<br>Patienten har behov for et ekstra sæt hænder til nogle behandlinger (eks. fysiske handlinger) og er som sådan behandlet af en af ovennævnte faggrupper samt en assistent (som evt. kan være uuddannet).<br>Eller der er behov for et intensivt program med ≥ 25 timers behandling pr. uge, (eks. 4-5 timer per dag, 5 dage om ugen), hvoraf nogle kan være med en assistent eller gruppebehandling i tillæg til deres individuelle daglige program.
**TI 4**	**Meget højt niveau – meget intensivt** (eks. behov for 2 erfarne behandlere. eller i alt 1:1 behandling > 30 timer om ugen).<br>Patienten har et meget kompliceret behandlingsbehov, som kræver to erfarne behandlere af gangen (med eller uden en tredje assistent) – f.eks. til at håndtere komplicerede fysiske behov, håndtering af uønsket adfærd etc. – eller behov for et meget intensivt program, som involverer > 30 timers terapi pr. uge.
**Total**	**Samlet T-score (TD + TI) :............**

	**Behov for hjælpemidler:**<br>Beskriver behovet for personlige hjælpemidler	
**E 0**	Intet behov for hjælpemidler	
**E 1**	Behov for basale hjælpemidler	Kørestol<br>Trykaflastningspude<br>Speciel madras<br>Stå-støtte-bord<br>Standard skinne<br>Andet standardustyr:<br><br>…………………………………………………..
**E 2**	Behov for højt specialiseret udstyr (eks. Specialfremstillet udstyr som er tilpasset eller fremstillet til patienten)	Omverdenskontrol<br>Kommunikations- hjælpemiddel<br>Specialtilpasset sæde/kørestol<br>Specialtilpasset stand-hjælpemiddel<br>Specialtilpasset skinne<br>Assisteret respiration<br>Andet specialiseret hjælpemiddel:<br><br>……………………………………………

Den danske oversættelse baseres på RCS Version 13, Lynne Turner-Stokes 05.04.2012

Yderligere oplysninger kan fås ved at rette henvendelse til Thomas Maribo: thomas.maribo@stab.rm.dk

	Benyt kun den højeste af disse to								
CPR xxxxxx-xxxx	Grund-læggende pleje	Kognitive/ adfærdsmæs-sige behov	Sygepleje-faglige behov	Lægefaglige behov	Tværfaglige indsatser		Hjælpe-midler	Total RCS-E score	Kommentarer
	C 0-4	R 0-4	N 0-4	M 0-4	TD 0-4	TI 0-4	E 0-2	0-22	

Total RCS-E score: C eller R (brug den højeste score) + N + M + TD + TI + E
