# Supplementary material for: Assessment of primary rehabilitation needs in neurological rehabilitation: translation, adaptation and face validity of the Danish version of Rehabilitation Complexity Scale-Extended
Source: BMC Neurol. 2016 Oct 21;16:205. doi: 10.1186/s12883-016-0728-7 (PMC5073960; doi:10.1186/s12883-016-0728-7)
Supplement: Additional file 2: — How to assess the English version of RCS-E. (PDF 100 kb) [file 12883_2016_728_MOESM2_ESM.pdf]

English version of Rehabilitation Complexity Scale-Extended

Rehabilitation Complexity Scale-Extended, English version is published in reference 7 and 8 as noticed in the paper

7. Turner-Stokes L, Disler R, Williams H. The Rehabilitation Complexity Scale: a simple, practical tool to identify 'complex specialised' services in neurological rehabilitation. Clin Med. 2007;7: 593-599.
8. Turner-Stokes L, Scott H, Williams H, Siegert R. The Rehabilitation Complexity Scale--extended version: detection of patients with highly complex needs. Disabil Rehabil. 2012;34: 715-720. doi: 10.3109/09638288.2011.615880 [doi].

The RCS-E is also available from King's College London
http://www.kcl.ac.uk/lsm/research/divisions/cicelysaunders/resources/tools/rcse.aspx. Follow the link "To obtain the clinical tool, please contact the UKROC team."
